# Development of Gamma-Aminobutyric Acid (GABA)-Rich and Probiotic-Fermented Soymilk Using *Lactiplantibacillus plantarum* W12 as a Starter Culture

**DOI:** 10.17113/ftb.64.02.26.9241

**Published:** 2026-06-15

**Authors:** Nguyen Cao Cuong, Bir Bahadur Thapa, Ngoc Hieu Nguyen, Tran Thanh Quynh Anh, Pham Hoang Son Hung, Peter Vandamme, Do Thi Bich Thuy

**Affiliations:** 1 Faculty of Engineering and Food Technology, University of Agriculture and Forestry, Hue University, 102 Phung Hung Street, Thua Thien Hue 530000, Vietnam; 2 Molecular Biotechnology Laboratory, School of Biotechnology, Institute of Agricultural Technology, Suranaree University of Technology, 111 University Avenue, Nakhon Ratchasima 30000, Thailand; 3 University of Medicine and Pharmacy at Ho Chi Minh City, 217 Hong Bang Street, District 5, Ho Chi Minh City 700000, Vietnam; 4 Faculty of Animal Sciences and Veterinary Medicine, University of Agriculture and Forestry, Hue University, 102 Phung Hung Street, Thua Thien Hue 530000, Vietnam; 5 Laboratory of Microbiology, Department of Biochemistry and Microbiology, Ghent University, K.L. Ledeganckstraat 35, 9000 Ghent, Belgium; 6 Institute of Research and Development, Duy Tan University, 254 Nguyen Van Linh Street, Da Nang 50312, Vietnam; 7 School of Engineering and Technology, Duy Tan University, 254 Nguyen Van Linh Street, Da Nang 50312, Vietnam

**Keywords:** gamma-aminobutyric acid, fermented soymilk, *Lactiplantibacillus plantarum*, response surface methodology, glutamate decarboxylase, functional food

## Abstract

**Research background:**

Fermented soymilk has emerged as a potential functional food due to its nutritional and health-promoting properties. Enhancing its functionality by enriching it with gamma-aminobutyric acid (GABA), a neurotransmitter with various health benefits, is an area of active research. This study aims to develop a novel GABA-enriched fermented soymilk using a newly isolated *Lactiplantibacillus plantarum* strain with both probiotic and GABA-producing capabilities.

**Experimental approach:**

Five *L. plantarum* strains isolated from Vietnamese soybean whey were screened for GABA production and probiotic characteristics. Strain W12, which exhibited superior performance, was selected for optimisation. Response surface methodology with a central composite design was used to optimise monosodium glutamate (MSG) and sucrose amounts for maximal GABA yield. A time-course study was then conducted to monitor bacterial growth, pH changes, organic acid production, and GABA accumulation during fermentation under the optimised conditions.

**Results and conclusions:**

*L. plantarum* W12 demonstrated exceptional probiotic traits: 97.1 % survival at pH=2.5, 96.5 % survival in bile salts/pancreatin, 97.3 % pepsin tolerance, 96.7 % auto-aggregation and 85.4 % hydrophobicity in chloroform. Initial GABA production reached (9.1±0.4) mM. Response surface optimisation predicted a maximum GABA concentration of 34.2 mM at 1.564 mg/mL MSG and 10.93 % sucrose, which was experimentally validated at (34.5±1.0) mM (15 h, 45 °C). Lactic acid was the predominant organic acid produced ((182.4±8.0) mg/g at 18 h), with viable cell counts exceeding 7.9 log CFU/mL, meeting probiotic thresholds.

**Novelty and scientific contribution:**

This study presents the first comprehensive characterisation of *L. plantarum* W12, a strain combining exceptional GABA biosynthesis with robust probiotic properties. The systematic response surface methodology (RSM) optimisation framework and detailed metabolic profiling provide reproducible protocols for developing multifunctional fermented soy beverages with applications in neurological and gastrointestinal health promotion.

## INTRODUCTION

Lactic acid bacteria (LAB) are important microorganisms in industry and have a key role as starter cultures in many fermented food products. They convert carbohydrate compounds into lactic acid as the main end product of fermentation. In addition, they enhance the quality of fermented food by creating flavour and aroma through the transformation of lipids and proteins into esters, alcohols and organic acids (*1*). LAB in the intestinal tract produce essential vitamins (*e.g*. thiamine, niacin, folic acid, pyridoxine, vitamin B12) and beneficial enzymes like lactase, and release free amino acids and short-chain fatty acids (*2*). These beneficial bacteria also contribute to gut health by inhibiting the growth of pathogenic bacteria through competitive exclusion and reducing the risk of diarrhoea.

Recently, LAB have attracted attention for their ability to produce gamma-aminobutyric acid (GABA), a non-proteinaceous amino acid present in the human body (*3*), and a neurotransmitter crucial for different functions. This GABA-producing capacity makes LAB fermentation a promising approach for developing functional foods with enhanced health benefits. GABA exerts multiple physiological effects in the human body, including regulation of blood pressure through vasodilation (*4*), reduction of anxiety and stress by modulating neural excitability (*5*), improvement of sleep quality, enhancement of immune function, and potential benefits in managing diabetes through improved insulin secretion and glucose metabolism (*6*). Recent studies have also demonstrated the role of GABA in maintaining intestinal homeostasis, where it attenuates ischaemia-reperfusion-induced alterations in intestinal immunity through increased IgA secretion and enhanced antioxidant activity (*7*). These diverse health-promoting effects make GABA-enriched functional foods particularly attractive for preventive nutrition and wellness applications.

Soybeans, rich in antioxidants like phenolics and isoflavones, are recognised as a potential functional food and offer benefits such as cardiovascular protection, antidiabetic effects, antioxidant properties, anticancer properties, and blood pressure regulation (*8*). Soymilk, a soybean extract, is a nutritious food containing high-quality protein, unsaturated fatty acids, lecithin, and isoflavones, while being cholesterol-free and lactose-free, making it suitable for individuals with lactose intolerance (*9*). As a plant-based alternative to cow's milk yoghurt, fermented soymilk offers health benefits, including protection against cancer and diabetes, improved memory, and enhanced wound healing (*10*).

Recently, LAB fermentation of soymilk has attracted scientific interest. Studies have shown that fermentation with *Lactiplantibacillus plantarum* Y16 increases the antioxidant capacity of soymilk (*11*). Research has also focused on the influence of added carbohydrates on the volatile organic compound profile of fermented soymilk, affecting sensory characteristics (*12*). Furthermore, fermentation with *L. brevis* and *L. plantarum* has been shown to alter soymilk composition, reducing isoflavones while increasing aglycones, ornithine, enzyme activity (pancreatic lipase, α-amylase, α-glucosidase) and antioxidant capacity (*13*). Studies have also explored the impact of different LAB strains, such as *Lactobacillus fermentum* SMN10-3(A) and *Lactococcus lactis* SMN15-6(B), on GABA content and flavour of fermented soymilk, and revealed that combining strains can enhance the GABA yield (*3*).

Supplementing soymilk with cell-free supernatant from *Lactiplantibacillus plantarum* BC112 has been shown to significantly enhance GABA production by *Enterococcus faecium* through quorum sensing modulation and upregulation of glutamate decarboxylase (GAD) gene expression (*14*). The cell-free supernatant contains secreted metabolites and signalling molecules that can influence the metabolic activity and gene expression of co-cultured GABA-producing strains, thereby increasing overall GABA yield in fermented products. Optimisation of medium composition and culture conditions to maximise GABA yield in soymilk fermented with *L. plantarum* Lp3, including the investigation of the effects of strain ratios, monosodium glutamate (MSG) concentration, fermentation time, and temperature, has been conducted using response surface methodology (*15*).

Among LAB, *L. plantarum* is particularly attractive due to its diverse health-promoting properties, including probiotic potential, antioxidant, anti-obesity, anticancer, and antidiabetic effects (*16*). This study aims to develop a novel symbiotic soymilk that is enriched in GABA and has probiotic properties, using *L. plantarum* W12. This strain was isolated from soybean whey and selected for its high GABA production and probiotic potential. We investigated the optimisation of MSG and sucrose supplementation to maximise GABA production in the fermented soymilk. Additionally, we analysed changes in pH, GABA content, organic acids, and LAB viability during fermentation to understand the metabolism of *L. plantarum* W12 during soymilk fermentation.

## MATERIALS AND METHODS

### Samples

Samples (*N*=20) were collected from soybean whey obtained from five traditional tofu manufacturers in Hue City, Vietnam, located in Thuan Hoa, Phu Xuan, Vi Da, An Cuu, and Kim Long Wards, during March–April 2023. Tofu was made using traditional methods: soaking whole soybeans (local Vina variety) for 8-12 h, grinding with water (1:8 ratio), filtering to obtain soymilk, and coagulating with 20–22 % whey from the previous batch (pH=4.0-4.5). The soybean whey, a byproduct of tofu coagulation, was collected immediately after pressing and transported on ice to the laboratory within 2 h of production. Samples were assigned unique identification codes (TW-01 to TW-20) and stored at −80 °C until bacterial isolation.

### Isolation of lactic acid bacteria

A mass of 15 g of each soybean whey sample was aseptically ground and homogenised in 135 mL of Ringer’s solution (Sigma-Aldrich, Merck, Milan, Italy) using a stomacher (Bag Mixer 400; Interscience, Saint Nom, France) at maximum speed for 2 min. Serial decimal dilutions of the homogenate were plated onto de Man, Rogosa, and Sharpe (MRS) agar (Oxoid, Milan, Italy) and incubated anaerobically at 37 °C for 48 h. Fifteen to twenty colonies per sample were randomly selected and purified by repeated streaking on MRS agar. Gram staining and catalase testing were performed on all isolates. Gram-positive and catalase-negative isolates were tentatively identified as LAB and stored in Microbank™ vials (Pro-Lab Diagnostics, Richmond Hill, ON, Canada) at -80 °C until further analysis.

### Pathogenic indicator strains

The pathogenic indicator strains used in this study were obtained from standard culture collections to ensure reproducibility and biosafety. The following strains were used: *Escherichia coli* ATCC 25922 (American Type Culture Collection, Manassas, VA, USA), *Staphylococcus aureus* ATCC 25923, and *Salmonella* Typhimurium ATCC 14028. All strains were maintained on tryptic soy agar (TSA, Oxoid) at 4 °C with monthly subculturing. Working cultures were prepared fresh before each assay by overnight incubation in tryptic soy broth (TSB, Oxoid) at 37 °C.

### MALDI-TOF MS analysis

Putative LAB isolates were identified using matrix-assisted laser desorption/ionization time-of-flight (MALDI-TOF) mass spectrometer (MALDI Biotyper microflex LT/SH; Bruker Diagnostics/Microbiology, Bremen, Germany) equipped with MBT Compass Explorer software v. 4.1. Spectral matching was performed against the commercial Bruker Automated Library (BDAL) Mass Spectrum Profile (MSP-11897) and the proprietary Laboratory of Microbiology, Ghent University (LM UGENT ID - MSP-6102). Following the manufacturer's protocols, fresh colonies were transferred directly to MALDI target plates using the direct transfer method. A volume of 1 μL of 70 % formic acid was added, followed by 1 μL of HCCA matrix solution (α-cyano-4-hydroxycinnamic acid in 50 % acetonitrile and 2.5 % trifluoroacetic acid). Spectra were acquired in positive linear mode (*m*/*z*=2000–20 000). Score values ≥2.0 indicated highly probable species-level identification, scores 1.7-1.99 indicated reliable genus-level identification, and scores <1.7 were considered unidentified.

### Preparation of inoculants

LAB strains were activated by culturing in MRS broth (Sigma-Aldrich, Merck) at 37 °C for 24 h. Cells were harvested by centrifugation at 10 000×*g* and 4 °C for 5 min (centrifuge 5424R; Eppendorf AG, Hamburg, Germany). The pellets were washed and resuspended in peptone water to achieve a final number of 8–9 log CFU/mL using the absorbance at 600 nm (BioSpectrometer fluorescence; Eppendorf, Hamburg, Germany) as a standard for cell density. This suspension served as the inoculum for soymilk fermentation.

### Soymilk fermentation

Soymilk (100 mL, 4 % protein content) was used as the base medium for all fermentation experiments. The soymilk was supplemented with MSG and/or carbon sources as described below, and inoculated individually with each of the five selected LAB strains (*L. plantarum* W1, W3, W4, W5 and W12) at an initial value of 8 log CFU/mL.

#### Initial GABA screening

For initial screening of GABA-producing capacity, the five selected *L. plantarum* strains (W1, W3, W4, W5 and W12) were cultured in MRS broth supplemented with 1 % (*m*/*V*) MSG at 37 °C for 24 h.

#### MSG amount optimisation

To determine the optimal MSG concentration for GABA production by *L. plantarum* W12 in soymilk, MSG was added at 0, 0.5, 1.0, 1.5 and 2.0 % (*m*/*V*). Fermentation temperature was increased to 45 °C for final optimisation to reduce fermentation time to 15 h while maintaining bacterial viability. GABA concentration was measured by high-performance liquid chromatography (HPLC; Shimadzu Scientific Instruments, Columbia, MD, USA).

#### Carbon source screening

To evaluate the effect of different carbon sources on GABA production, soymilk supplemented with 1.5 % (*m*/*V*) MSG was further supplemented with 5 % (*m*/*V*) of individual carbon sources: lactose, sucrose, glucose, or maltose. A control without additional carbon source was also prepared. Each supplemented soymilk was inoculated with *L. plantarum* W12 and fermented at 43 °C for 15 h. GABA concentration was determined by HPLC (Shimadzu Scientific Instruments).

#### Sucrose amount optimisation

Based on the carbon source screening results, the optimal sucrose amount was investigated. Soymilk containing 1.5 % (*m/V*) MSG was supplemented with sucrose at 0, 5, 10, 15 and 20 % (*m*/*V*). Fermentation conditions and GABA analysis were performed as described above.

#### Time-course fermentation study

For final product characterisation, *L. plantarum* W12 was inoculated into soymilk containing optimised concentration of MSG (1.564 mg/mL) and amount of sucrose (10.93 %, *m*/*V*) at an initial cell value of 5·10^6^ CFU/mL. Fermentation was conducted at 43 °C with an initial pH=6.0. Samples were collected at 0, 3, 6, 9, 12, 15 and 18 h for analysis of cell growth, pH, GABA concentration, and organic acid production.

### Enumeration of viable cells

Viable cell counts were determined using the pour plate method. For fermented soymilk samples, 20 g of sample were homogenised with 180 mL of sterile diluent containing 0.1 % (*m*/*V*) Bacto™ peptone (Difco Laboratories, Detroit, MI, USA) and 0.9 % (*m*/*V*) NaCl. For liquid samples (inocula and fermentation broths), samples were directly diluted in sterile 0.85 % (*m*/*V*) NaCl solution. Serial tenfold dilutions were prepared, and 1-mL aliquots of appropriate dilutions were plated onto MRS agar in duplicate using the pour plate technique. Plates were incubated anaerobically at 37 °C for 48 h. Viable cells were enumerated according to [REMOVED HYPERLINK FIELD]standard methods. Results are expressed as log_10_ CFU/mL for liquid samples or log CFU/g for fermented soymilk and represent the mean of three independent experiments.

### Chemical analysis by HPLC

#### Sample preparation

Two different sample preparation methods were used depending on the analyte and sample matrix. For fermented soymilk, the extraction procedure was adapted from Costa *et al.* (*17*). Briefly, 4 g were accurately weighed and mixed with 5 mL of HPLC-grade water (Sigma-Aldrich, Merck). This mixture was then further diluted with 25 mL of HPLC-grade acetonitrile (Sigma-Aldrich, Merck). The resulting solution was filtered through a 0.45-µm membrane filter to remove any particulate matter. An aliquot of 500 µL of the filtrate was then used for HPLC analysis.

For MRS broth cultures, LAB strains were cultivated in MRS broth. The supernatants were collected after centrifugation at 10 000×*g* and 4 °C for 15 min (centrifuge 5424R; Eppendorf AG). Proteins were precipitated by adding 3 % (*m/V*) sulfosalicylic acid, followed by a second centrifugation step. The resulting protein-free supernatant was then used for GABA derivatisation, as described in section GABA quantification.

#### Organic acid analysis

Organic acids were analysed using an HPLC system (Shimadzu Scientific Instruments) equipped with an OA-1000 organic acid column (6.5 mm×300 mm; Alltech Associates Inc., Columbia, MD, USA) and a UV detector set at 210 nm. The mobile phase consisted of 0.005 M H_2_SO_4_ at a flow rate of 0.4 mL/min, and the column temperature was maintained at 35 °C. Reference organic acids (Sigma-Aldrich, Merck) were used for calibration and quantification.

#### GABA quantification

GABA was analysed following a method adapted from Thuy *et al.* (*18*). Briefly, GABA in the prepared samples was derivatised with 4 mM 4-dimethylaminoazobenzen-4’-sulfonyl chloride (dabsyl chloride) for soymilk and MRS broth samples. Dabsyl-GABA was quantified at *λ*=465 nm using a Shimadzu LC-20A HPLC system equipped with an SPD-20A UV-Vis detector. Separation occurred on a Supelco C18 column (250 mm×4.6 mm i.d., 5 μm particle size) maintained at 55 °C. The mobile phase consisted of an isocratic mixture of 25 mM ammonium acetate (0.1 % acetic acid) and acetonitrile (*φ*(acetonitrile)=74 %) at a flow rate of 1 mL/min. GABA concentrations were determined using a calibration curve in the range 0–10 mM.

### Assessment of probiotic properties

Following the method by Li *et al.* (*19*), the following probiotic properties were analysed.

#### Low pH tolerance assay

LAB cells were harvested from overnight cultures by centrifugation (10 000×*g*, 4 °C, 15 min, centrifuge 5424R; Eppendorf AG) and resuspended in a sterile solution of 0.1 % Bacto peptone (Difco Laboratories) and 0.9 % (*m*/*V*) NaCl. A volume of 1 mL of cell suspension was then inoculated into MRS broth and adjusted to pH=2.5. The initial absorbance (*A*_T0_) was measured at 600 nm. Following incubation at 37 °C for 4 h, the absorbance was measured again (*A*_T4_). Survival percentage (H/%) was calculated using the following equation:


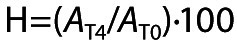
 /1/

#### Bile salts and pancreatin tolerance assay

LAB cells from overnight cultures in MRS broth were harvested by centrifugation (10 000×*g*, 4 °C, 15 min, centrifuge 5424R; Eppendorf AG) and resuspended. A volume of 1 mL of cell suspension was inoculated into a sterile saline solution (0.85 % NaCl, *m*/*V*) containing 1 mg/mL pancreatin (Sigma-Aldrich, Merck, Seoul, South Korea) and 0.3 % (*m*/*V*) bile salts (MilliporeSigma, Merck KGaA, Darmstadt, Germany) The solution was incubated at 37 °C. Absorbance was measured at the beginning (*A*_T0_) and after 6 h of inoculation (*A*_T6_). Tolerance was calculated as survival percentage (H/%), using the following equation:


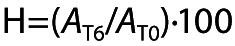
 /2/

#### Pepsin tolerance assay

After 24 h of incubation, LAB cultures were centrifuged (400×*g*, 4 °C, 6 min, centrifuge 5424R; Eppendorf AG). Simulated gastric fluid was prepared by adding pepsin (Sigma-Aldrich, Merck, Burlington, MA, USA) to a 0.85 % NaCl solution to achieve a final concentration of 3 mg/mL and adjusting the pH to 2.5. The cell pellet was resuspended in the simulated gastric fluid, vortexed, and the initial absorbance was measured at 600 nm (*A*_T0_). After incubation at 37 °C for 4 h, the absorbance was measured again (*A*_T4_). Survival percentage (H/%) was calculated using Eq. 1.

#### Antibiotic susceptibility assay

Antibiotic susceptibility was determined using the disc diffusion method of Oh *et al.* (*20*) and interpreted according to the guidelines established by the European Food Safety Authority (EFSA) for *Lactiplantibacillus* species (*21*). The following antibiotic discs were placed on agar plates containing bacteriological grade agar powder (GRM026; HiMedia, Mumbai, India) inoculated with the lactic acid bacteria (LAB) strains (in µg): vancomycin 30, clindamycin 2, tetracycline 30, ampicillin 10, streptomycin 10, penicillin 10, chloramphenicol 30, erythromycin 15, kanamycin 30 and gentamicin 10. After 24 h of incubation at 37 °C, the diameters of the inhibition zones were measured.

#### Aggregation properties

Auto-aggregation: Following the method of Li *et al.* (*19*), overnight LAB cultures were centrifuged (10 000×*g*, 4 °C, 10 min, centrifuge 5424R; Eppendorf AG), and the pellets were resuspended in 1 mL of phosphate-buffered saline (PBS). Initial absorbance (*A*_0_) was measured at 600 nm. After incubation at 37 °C for 20 h, the absorbance of the upper suspension (*A*_20_) was measured. Autoaggregation (%) was calculated using the following equation:


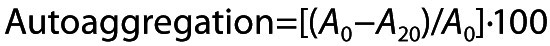
 /3/

Co-aggregation: LAB cells from 24-hour cultures in MRS broth were mixed with equal volumes (1.5 mL) of pathogenic strains (*Staphylococcus aureus, Escherichia coli* and *Salmonella *Typhimurium). The mixtures were incubated at 37 °C for 5 h. The absorbance of the supernatants (*A*_mix_) was measured at 600 nm. The absorbance of the individual LAB (*A*_probiotic_) and pathogen (*A*_pathogen_) suspensions was also measured. Co-aggregation (%) was calculated using the following equation:


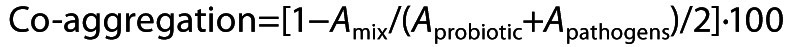
 /4/

#### Hydrophobicity assay

LAB cultures grown in MRS broth at 37 °C for 24 h were centrifuged (400×*g*, 4 °C, 10 min, centrifuge 5424R; Eppendorf AG). The pellets were resuspended in 0.1 M KNO_3_. The initial absorbance (*A*_0_) was measured at 600 nm. Cell suspensions of 2 mL were mixed with 1 mL of organic solvent (chloroform, ethyl acetate or xylene). After incubation at room temperature for 10 min and vortexing for 2 min, the mixture was left undisturbed for 20 min to allow complete phase separation, and the absorbance of the aqueous phase (*A*_1_) was then measured. Cell surface hydrophobicity (%) was calculated using the equation below according to Oh *et al.* (*20*):


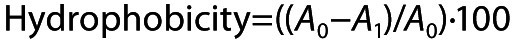
 /5/

#### Evaluation of antimicrobial activity

The antimicrobial activity was determined using the agar spot method of Hernandez *et al.* (*22*). Pathogen indicator strains included *S. aureus, S. *Typhimurium and *E. coli*. A suspension of LAB cells was prepared by mixing the overnight LAB biomass in 0.85 % NaCl. This suspension (5 µL) was dropped onto MRS agar surfaces, allowed to dry, and grown at 37 °C for 18 h. Cells of pathogenic bacteria were suspended in 1 % agar at approx. 37 °C. These agar suspensions were immediately poured onto the spot-inoculated MRS agar plates, left to solidify, and then incubated at 37 °C for 24–48 h. The diameters of the clear zones of inhibition of pathogenic bacteria around the spots were determined using a Vernier calliper. Positive controls consisted of MRS agar plates inoculated with known antimicrobe-producing *L. plantarum* reference strain (ATCC 14917), while negative controls consisted of uninoculated MRS agar spots overlaid with pathogen-containing agar. Inhibition zones were measured only when they exceeded 2 mm outside the LAB colony margin to ensure specific antimicrobial activity.

### Optimisation using response surface methodology

Central composite design (CCD) of response surface methodology (RSM) was used to optimise the concentrations of MSG and sucrose to maximise GABA production. The two independent variables were X_1_: MSG concentration (mg/mL) and X_2_: sucrose amount (%, *m*/*V*). The experimental design consisted of 13 runs, including 4 factorial points, 4 axial points for a rotatable design, and 5 centre points. The two independent variables were studied at three different levels (Table 1).

**Table 1:** Ranges of the two independent variables used in response surface methodology (RSM)

Independent variable		Level				
		−α	−1	0	1	+α
X_1_	*γ*(MSG)/(mg/mL)	0.79	1.00	1.50	2.00	2.21
X_2_	(*m*(sucrose)/*V*(solution))/%	2.93	5.00	10.00	15.0	17.07

The coded values were transformed into actual values (Table 2) using the following equations:


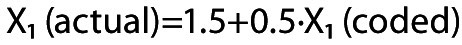
 /6/


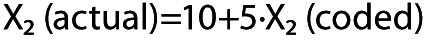
 /7/

**Table 2:** Experimental plan for optimisation of γ-aminobutyric acid (GABA) yield using response surface methodology (RSM)

Experiment	Variable (coded)		Variable net value		*Y*(GABA)/mM	
	X_1_	X_2_	X_1_	X_2_	Actual	Predicted
1	-1	-1	1.00000	5.000	17.82	17.30
2	1	-1	2.00000	5.000	16.33	18.83
3	-1	1	1.00000	15.000	27.28	25.96
4	1	1	2.00000	15.000	15.77	17.46
5	-1.41421	0	0.79289	10.000	19.12	20.66
6	1.41421	0	2.20711	10.000	18.45	15.73
7	0	-1.41421	1.50000	2.9289	20.16	19.00
8	0	1.41421	1.50000	17.0711	24.19	24.16
9	0	0	1.50000	10.0000	34.00	33.96
10	0	0	1.50000	10.0000	32.98	33.96
11	0	0	1.50000	10.0000	33.59	33.96
12	0	0	1.50000	10.0000	34.11	33.96
13	0	0	1.50000	10.0000	35.10	33.96

The experiment was conducted randomly. X_1_=monosodium glutamate (*γ*(MSG)/(mg/mL)), X_2_=(*m*(sucrose)/*V*(solution))/%

The second-order polynomial model was:


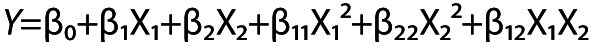
 /8/

where *Y* represents the predicted GABA yield (mM), X_1_ and X_2_ represent the coded values of MSG concentration and sucrose amount, β_0_ is the intercept term, β_1_ and β_2_ are the linear coefficients, β_11_ and β_22_ are the quadratic coefficients, and β_12_ is the interaction coefficient between MSG and sucrose.

### Statistical analysis

Response surface methodology (RSM) was used to analyse the experimental data and fit a second-order polynomial model (*23*). Design-Expert software v. 12.0.3.0 was used for the experimental design, data analysis, and optimisation procedures (*24*). Model adequacy was evaluated using analysis of variance (ANOVA), considering the model F-value, lack-of-fit test, and coefficient of determination (R^2^). The significance of model terms was determined using p-values (α=0.05), with only significant terms included in the reduced models. Non-significant linear terms were retained if the corresponding quadratic or interaction terms were significant, following established hierarchical model reduction principles. All experiments were performed in triplicate, and the results are presented as mean value±standard deviation. One-way ANOVA and Tukey's HSD *post-hoc* test were used to compare the mean values, with statistical significance set at p≤0.05.

## RESULTS AND DISCUSSION

### Isolation and identification of L. plantarum strains

Assessment of the absence of catalase activity and Gram-positive staining yielded 30 presumptive LAB isolates. Five isolates with GABA-producing capability (see below), namely W1 (R-49778), W3 (R-49768), W4 (R-49769), W5 (R-49770) and W12 (R-49779), were identified as *Lactiplantibacillus plantarum* by MALDI-TOF MS with high score values.

### GABA production optimisation by L. plantarum

**Fig. 1:**
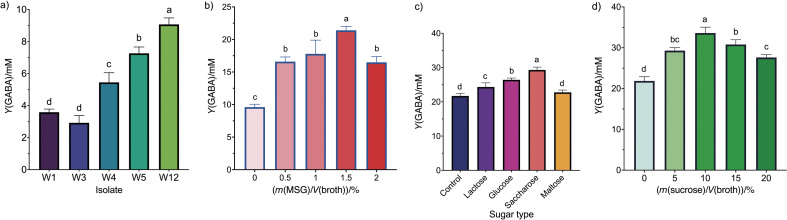
Optimisation of γ-aminobutyric acid (GABA) production by *Lactiplantibacillus plantarum* strains: a) production capacity of five *L. plantarum* isolates (W1, W3, W4, W5 and W12) in MRS broth supplemented with 1 % MSG after 24 h of fermentation at 37 °C, b) effect of MSG amount (0–2 %, *m/V*) on GABA production by *L. plantarum* W12 in soymilk fermented at 43 °C for 15 h, c) effect of 5 % (*m/V*) carbon source type (lactose, sucrose, glucose and maltose) on GABA production by *L. plantarum* W12 in soymilk containing 1.5 % MSG, d) effect of sucrose amount (0–20 %, *m/V*) on GABA production by *L. plantarum* W12 in soymilk containing 1.5 % MSG. Data are presented as mean±standard deviation (*N*=3). Different letters indicate significant differences (p≤0.05, Duncan's multiple range test)

Fig. 1 shows the optimisation of GABA production by *L. plantarum* strains. The GABA production capacity of the five selected *L. plantarum* strains was assessed (Fig. 1a). *L. plantarum* W12 exhibited the highest GABA production ((9.1±0.4) mM), significantly higher than the other isolates (p≤0.05). *L. plantarum* W5 also showed substantial GABA production ((7.3±0.4) mM). Isolates W1, W3 and W4 produced lower GABA concentrations ((3.6±0.2), (2.9±0.5) and (5.4±0.6) mM, respectively).

GABA production varied significantly among *L. plantarum* strains, likely due to differences in genetic background, glutamate decarboxylase (GAD) activity, and associated metabolic pathways (*25*). Among them, *L. plantarum* W12 showed the highest GABA yield and was selected for further development of GABA-enriched fermented soymilk.

The initial GABA production by *L. plantarum* W12 in MRS broth ((9.1±0.4) mM at 24 h with 1 % MSG) was lower than that reported by Harnentis *et al.* (*26*) (211.17 mM under fully optimised conditions: pH=5.5, 36 °C, 500 mM glutamate (50× higher substrate than in our study), 84 h fermentation (5.6× longer) with yeast extract and glucose supplementation), but fell within the range reported by Ledashcheva *et al.* (*27*) (7.03–54.21 mM in MRS broth with 1 % MSG, with strain GB111 achieving 54.21 mM) and was lower than the optimised value by Thuy *et al.* (*18*) (25.52 mM under optimised conditions: 5·10^6^ CFU/mL, 2 % MSG, pH=7, 35 °C, 48 h in MRS broth). More importantly, under optimised fermentation conditions in soymilk, W12 achieved a maximum GABA yield of (34.5±1.0) mM (*i.e*. 3.6 g/L), which was substantially higher than the 1.76 mg/mL (17.07 mM) reported by Xia *et al.* (*3*) in co-fermented soymilk using *L. fermentum* SMN10-3(A) and *L. lactis* SMN15-6(B) at a ratio of 2:1. This shows that *L. plantarum* W12 exhibits superior GABA-producing capacity, specifically in soymilk fermentation compared to previously reported strains.

### Probiotic potential of L. plantarum strains

This study evaluated the probiotic potential of *L. plantarum* strains W1, W3, W4, W5 and W12 by assessing their gastrointestinal tolerance, autoaggregation and co-aggregation abilities, cell surface hydrophobicity, and antagonistic effects against pathogenic bacteria. As described below, *L. plantarum* W12 exhibited the most promising probiotic characteristics and was subsequently selected for soymilk fermentation studies.

#### Gastrointestinal tolerance

Survival under simulated gastrointestinal conditions is crucial for probiotic efficacy. All tested strains showed considerable tolerance to low pH, bile salts, pancreatin and pepsin (Table 3). *L. plantarum* W3 and W12 exhibited the highest survival rates in low pH and bile salt/pancreatin conditions (over 95 and 96 % survival, respectively). While all strains showed good pepsin tolerance (above 84 %), W1 and W12 had the highest survival rates, exceeding 97 %. These results suggest that these strains possess robust resistance to the harsh conditions of the gastrointestinal tract. Acid tolerance is a critical probiotic trait, enabling survival through the stomach. At low pH (<4.5), proton influx requires substantial ATP for homeostasis, potentially leading to metabolic disruption and cell death (*28*). Gastric pH can drop to 1–2 during fasting (*29*). Thus, a cutoff of pH=3.0 for 3 h was used to simulate gastric conditions. All assessed *L. plantarum* strains survived well at pH<3.0. Bile tolerance is also essential for probiotic efficacy. Bile salts exert antimicrobial effects by disrupting bacterial membranes (*30*).

**Table 3:** Comprehensive probiotic properties of *Lactiplantibacillus plantarum* strains isolated from soybean whey

**Survival** **Strain**	**Gastrointestinal tolerance** **(Survival/%)**			**Hydrophobicity/%**			***d*(antagonistic zone)/mm**			**Auto-aggregation/%**	**Coaggregation with pathogenic bacteria/%**		
Low pH	Bile/pancreatin	Pepsin	Xylene	Chloroform	Ethyl acetate	*E. coli*	*S. aureus*	*S. *Typhimurium	*L. plantarum*	*E. coli*	*S. aureus*	*S. *Typhimurium
*L. plantarum* W1	(92.0±3.4)^c^	(89.3±2.0)^b^	(97.7±1.3)^a^	(9.79±0.29)^a^	(68.3±2.8)^b^	(7.3±1.2)^b^	(2.7±0.6)^c^	(15.7±0.6)^c^	(12.0±1.0)^b^	(93.7±2.3)^a^	(6.7±0.64^b^	(11.4±1.0)^c^	(0.11±0.04)^d^
*L. plantarum* W3	(98.0±2.4)^a^	(95.2±3.2)^a^	(88.1±2.9)^b^	(0.16±0.03)^e^	(10.6±1.5)^d^	(3.7±1.2)^d^	(8.3±1.2)^a^	(26.7±1.5)^a^	(15.0±1.0)^a^	(87.1±1.9)^b^	(5.4±0.5)^c^	(2.7±0.5)^e^	(17.7±1.6)^a^
*L. plantarum* W4	(87.0±2.6)^d^	(93.1±1.3)^b^	(84.2±2.3)^c^	(5.47±0.56)^b^	(2.6±07)^e^	(5.8±1.0)^bc^	(2.7±0.6)^c^	(16.3±1.5)^c^	(5.3±0.6)^d^	(83.2±1.8)^c^	(0.19±0.03)^d^	(18.3±0.9)^b^	(0.7±0.2)^d^
*L. plantarum* W5	(93.1±1.2)^bc^	(95.3±1.6)^a^	(87.9±1.2)^b^	(3.96±0.22)^c^	(16.7±1.0)^c^	(4.4±0.5)^cd^	(5.0±1.0)^b^	(20.7±1.5)^b^	(7.0±1.0)^c^	(87.3±1.0)^b^	(4.8±0.60^c^	(8.8±1.1)^d^	(11.4±1.4)^c^
*L. plantarum* W12	(97.1±2.0)^ab^	(96.5±1.0)^a^	(97.3±1.7)^a^	(2.87±0.43)^d^	(85.4±0.6)^a^	(12.7±0.6)^a^	(3.0±1.0)^c^	(28.0±1.0)^a^	(14.3±0.6)^a^	(96.7±1.8)^a^	(8.3±0.6)^a^	(20.8±1.6)^a^	(14.4±0.9)^b^

Values are presented as mean±standard deviation, *N*=3. Different letters in superscript within a column indicate significant differences (p≤0.05, Duncan's multiple range test)

#### Aggregation

Autoaggregation, the ability of bacterial cells to clump together, and coaggregation, the ability to adhere to pathogenic bacteria, are important for colonisation and competitive exclusion of pathogens in the gut. Table 3 shows that *L. plantarum* W12 had the highest autoaggregation ability (96.67 %), significantly higher than the other strains. While W12 also showed strong coaggregation with *S. aureus* (20.82 %) and *S. *Typhimurium (14.39 %), W3 demonstrated the highest coaggregation with *S. *Typhimurium (17.74 %). These varying co-aggregation profiles suggest strain-specific interactions with different pathogens.

Probiotic activity is also linked to the ability to aggregate and adhere to host tissues. Autoaggregation and coaggregation help form a barrier against pathogens (*31*). These traits were evident in the *L. plantarum* strains tested, supporting their probiotic potential (*32*).

#### Hydrophobicity

Cell surface hydrophobicity affects the adherence of probiotic bacteria to intestinal epithelial cells, contributing to colonisation. Table 3 (hydrophobicity) shows that *L. plantarum* W12 exhibited the highest hydrophobicity towards chloroform (85.45 %) and ethyl acetate (12.71 %), while W1 showed the highest hydrophobicity towards xylene (9.79 %). The variations in hydrophobicity across different solvents indicate differences in cell surface composition among the strains. Surface hydrophobicity correlates with aggregation and adhesion capabilities (*33*). Hydrophobicity was particularly high in strains W1 and W12, as assessed in chloroform. Bile exposure reduced hydrophobicity, likely affecting adhesion, which is consistent with previous findings (*34*).

#### Antagonistic effect

The ability to inhibit pathogenic bacteria is a key probiotic trait. *L. plantarum* W3 and W12 (Table 3, antimicrobial activity) showed the strongest antagonistic activity against all tested pathogens (*E. coli*, *Salmonella* and *S. aureus*), as indicated by the largest zones of inhibition. W12 notably exhibited the largest inhibition zones against *S. aureus* (28 mm) and *Salmonella* (14.33 mm), while W3 showed the strongest effect against *E. coli* (8.33 mm). These results highlight the potential of these strains to control the growth of common foodborne pathogens.

Based on the comprehensive evaluation of these probiotic properties, *L. plantarum* W12 emerged as the most promising candidate. Its superior performance in GABA production, combined with high gastrointestinal tolerance, autoaggregation, coaggregation with specific pathogens, and strong antagonistic activity, justifies its selection for further investigation in soymilk fermentation. This strain has significant potential for developing functional foods with enhanced probiotic benefits. The antibacterial activity of *L. plantarum* is well-documented (*35*), primarily through organic acid production and pH reduction (*36*). W12 showed antagonistic effects against *E. coli, S. aureus* and *S. *Typhimurium, likely due to its high GABA output and acidification. All tested strains produced significantly larger inhibition zones than negative controls (no inhibition zone), and positive controls using *L. plantarum* ATCC 14917 showed comparable inhibition patterns, validating the assay methodology.

The results presented here show that *L. plantarum* W12 successfully integrates two critical functional properties: exceptional GABA biosynthesis capacity and robust probiotic characteristics. The high survival rates of the strain under simulated gastrointestinal conditions (>96 % for pH, bile/pancreatin, and pepsin challenges, Table 3), strong auto-aggregation (96.67 %), and broad-spectrum antimicrobial activity against foodborne pathogens (inhibition zones of 28.00 mm against *S. aureus*, 14.33 mm against *Salmonella* and 3.00 mm against *E. coli*) provide confidence in its probiotic efficacy. The optimised GABA yield of 34.5 mM represents a 3.8-fold improvement over the initial screening value (9.1 mM) and positions this fermented soymilk among the highest GABA-containing plant-based fermented products reported to date.

The viable cell count exceeding 7.9 log_10_ CFU/mL at the end of fermentation ensures adequate probiotic dosage (typically ≥10^7^ CFU/mL for claimed health benefits) (*37*), while the high GABA concentration provides therapeutic potential for neurological and cardiovascular benefits. This dual functionality, combined with favourable sensory properties (mild acidity and clean flavour) and extended shelf life conferred by organic acid preservation, positions this product as a promising candidate for commercialisation as a functional, health-promoting beverage. Future research should focus on *in vivo* validation of GABA bioavailability and probiotic colonisation efficiency through animal models and clinical trials, as well as shelf-life stability studies and consumer acceptance testing to facilitate commercial development.

### Applications of L. plantarum W12 in soymilk fermentation

#### Factors influencing GABA biosynthesis

The initial cell count (5·10^6^ CFU/mL), temperature (45 °C), initial pH (7) and fermentation time (15 h) were kept constant to investigate the specific effects of MSG concentration and carbon source supplementation on GABA biosynthesis.

Additional MSG concentration in soymilk significantly affected GABA production by *L. plantarum* W12 (Fig. 1b). GABA production increased with MSG supplementation up to 1.5 % (*m/V*), reaching a maximum of (21.4±0.6) mM. However, increasing the MSG amount to 2 % did not further increase GABA production and showed a slight decrease, suggesting a potential inhibitory effect at higher concentrations or substrate saturation. This finding agrees with other studies reporting an optimal MSG concentration for GABA production in LAB fermentations (*38*). The decrease in GABA production at 2 % MSG could be due to substrate inhibition of glutamate decarboxylase or feedback inhibition mechanisms within the metabolic pathway (*39*). Therefore, 1.5 % MSG supplementation is optimal for maximising GABA production by *L. plantarum* W12 under these fermentation conditions. While high glutamate concentrations can enhance GAD activity (*40*), excessive amounts may disrupt metabolism *via* osmotic stress. Our data suggest that GABA production peaked at 15 h, followed by a decline, possibly due to substrate depletion, GABA degradation, or GAD feedback inhibition (*41*).

The results (Fig. 1c) indicate that the type of carbon source (lactose, sucrose, glucose or maltose) significantly affected GABA production. Supplementation with sucrose resulted in the highest GABA production ((29.3±0.8) mM), significantly exceeding all other treatments (p≤0.05). Glucose supplementation also led to a substantial increase in GABA ((26.4±0.5) mM), while lactose and maltose supplementation resulted in GABA concentrations comparable to the control (no added carbon source) ((24.3±1.2) and (22.8±0.7) mM, respectively).

The differences in GABA production with different carbon sources can be attributed to variations in their metabolic pathways and how efficiently they support the growth and metabolic activity of *L. plantarum* W12. Sucrose, a disaccharide composed of glucose and fructose, may provide a more sustained release of readily metabolisable sugars, leading to enhanced GABA production (*42*). The lower GABA concentrations observed with lactose and maltose could be due to differences in their uptake and utilisation by *L. plantarum* W12 or potential catabolite repression effects. Selecting an appropriate carbon source is therefore crucial for optimising GABA production in soymilk fermentation by *L. plantarum* W12.

Next, the optimal sucrose amount for GABA production was investigated. Varying amounts of sucrose (0, 5, 10, 15 and 20 %, *m/V*) were added to the MRS medium containing 1.5 % MSG. The results (Fig. 1d) of *L. plantarum* W12 demonstrate a clear influence of sucrose content on GABA production. GABA production increased with up to 10 % sucrose, reaching a maximum of (33.6±1.4) mM. However, further increases in sucrose content (15 and 20 %) resulted in decreased GABA production ((30.8±1.2) and (27.6±0.7) mM, respectively). This suggests that while sucrose enhances GABA production up to a certain point, excessive amounts can have a detrimental effect.

The decrease at higher amounts of sucrose could be attributed to factors including osmotic stress, substrate inhibition, and a shift in metabolic flux. High sucrose amounts can create osmotic stress for the bacteria, inhibiting their growth and metabolic activity (*43*). Excessive sucrose could potentially inhibit enzymes involved in GABA metabolism or related pathways. High sugar content might redirect metabolic flux away from GABA production towards other metabolic pathways, such as exopolysaccharide production or other stress responses (*44*). The results indicate that 10 % sucrose supplementation is optimal for maximising GABA production by *L. plantarum* W12 under these fermentation conditions. This finding is valuable for developing an efficient and cost-effective fermentation strategy for GABA-enriched soymilk production.

#### Optimisation of screened variables using central composite design

The MSG and sucrose amounts for enhanced GABA production by *L. plantarum* W12 in soymilk were optimised using a central composite design (CCD) of response surface methodology (RSM). The CCD comprised 13 experimental runs, including factorial, axial, and centre points (Table 2). The independent variables were MSG concentration (X_1_) and sucrose amount (X_2_), with GABA yield (*Y*, mM) as the response variable. GABA yield varied significantly depending on the concentrations of MSG and amount of sucrose.

A p<0.05 indicates that model terms are significant, while values greater than 0.10 indicate that the model terms are not significant. In the analysis of variance (*45*), the response surface model optimising GABA yield by *L. plantarum* W12 in soymilk was developed using a CCD of RSM with MSG concentration (X_1_) and sucrose amount (X_2_) as independent variables (Table 2). The quadratic model for GABA yield (*Y*, mM) was highly significant (F=39.65, p<0.0001, R^2^=96.59 %), with significant terms for MSG (p=0.0338), sucrose (p=0.0284), their interaction (p=0.0319), and quadratic terms for both MSG (p<0.0001) and sucrose (p<0.0001) (Table 4 and Table 5).

**Table 4:** Test of significance for regression coefficients

**Term**	**Coeff**	**SE coeff**	**T-value**	**P-value**	**VIF**
Constant	33.958	0.838	40.51	0.000	
A	-1.744	0.663	-2.63	0.034	1.00
B	1.824	0.663	2.75	0.028	1.00
A×A	-7.881	0.711	-11.09	0.000	1.02
B×B	-6.187	0.711	-8.71	0.000	1.02
A×B	-2.505	0.937	-2.67	0.032	1.00
Model summary					
Standard deviation of the residuals (s)			1.87427	Mean	25.30
R-squared (R^2^)			96.59 %	CV/%	7.41
Adjusted R-squared (Adj. R^2^)			94.15 %		
Predicted R-squared (Pred. R^2^)			77.61 %		

The results were obtained using the Design Expert software v. 12.0.3.0 (*24*). A=monosodium glutamate (*γ*(MSG)/(mg/mL)), B=(*m*(sucrose)/*V*(solution))/%. VIF=variance inflation factor, CV=coefficient of variation

**Table 5:** ANOVA for the quadratic model of γ-aminobutyric acid (GABA) yield

**Source**	**Sum of squares**	**df**	**Mean square**	**F-value**	**p-value**	
Model	696.47	5	139.29	39.65	< 0.0001	significant
A	24.33	1	24.33	6.93	0.0338	
B	26.62	1	26.62	7.58	0.0284	
AB	25.10	1	25.10	7.14	0.0319	
A^2^	432.03	1	432.03	122.99	< 0.0001	
B^2^	266.32	1	266.32	75.81	< 0.0001	
Residual	24.59	7	3.51			
Lack-of-fit	22.17	3	7.39	12.22	0.0175	significant
Pure error	2.42	4	0.6048			
Cor total	721.06	12				

The results were obtained using Design Expert software v. 12.0.3.0 (*24*). A=monosodium glutamate (*γ*(MSG)/(mg/mL), B=(*m*(sucrose)/*V*(solution))/%

The coded values for MSG (X_1_) and sucrose (X_2_) were converted to actual values using Eqs. 6 and 7.

The following second-order polynomial regression model was fitted to the data:


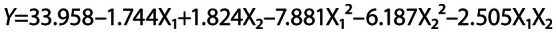
 /9/

where *Y* is the GABA yield (mM), and X_1_ and X_2_ are the coded values for MSG concentration and sucrose amount, respectively.

**Fig. 2:**
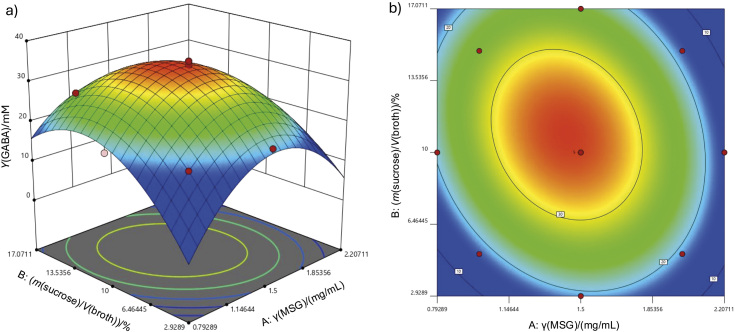
Response surface methodology optimisation of γ-aminobutyric acid (GABA) production by *Lactiplantibacillus plantarum* W12: a) three-dimensional response surface plot showing the combined effects of monosodium glutamate (MSG) concentration (mg/mL) and sucrose (%, *m/V*) amount on GABA yield (mM). The curved surface indicates a clear optimal region with maximum GABA production at intermediate levels of both variables, b) two-dimensional contour plot displaying the same optimisation landscape, where concentric rings represent GABA yield iso-response lines. The optimal conditions (1.564 mg/mL MSG and 10.93 % sucrose) are located at the centre of the highest contour region, predicting a maximum GABA yield of 34.24 mM

Response surface methodology (RSM) was used to visualise and optimise the combined effects of MSG (mg/mL) and sucrose (%, *m/V*) amounts on GABA yield (mM). The 3D and contour plots (Fig. 2a and Fig. 2b) showed a non-linear relationship between the independent variables and GABA yield, with a clear optimal region indicated by the curved surface and concentric rings, respectively. Model optimisation predicted a maximum GABA yield (*Y*_max_) of 34.2 mM at 1.6 mg/mL MSG and 10.9 % sucrose. These optimised conditions were experimentally validated. The optimisation results were: *Y*_max_=34.2 mM, X_1_(MSG)=1.6 mg/mL, and X_2_(sucrose)=10.9 %.

#### Time course study of cell growth, pH, organic acid and GABA content

A time course study was conducted to monitor changes in pH, cell growth, organic acid production, and GABA concentration during soymilk fermentation by *L. plantarum* W12 under optimised conditions (initial cell density 5·10^6^ CFU/mL, 45 °C, initial pH=6.0). Key factors influencing microbial GABA biosynthesis include cell density, pH, temperature, fermentation time and glutamate availability (*18*).

**Fig. 3:**
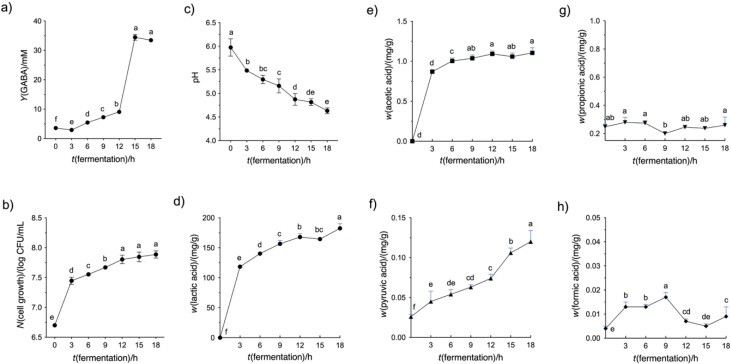
Time-course analysis of *Lactiplantibacillus plantarum* W12 soymilk fermentation under optimised conditions (1.564 mg/mL monosodium glutamate (MSG)), 10.93 % sucrose, 43 °C, initial pH=6.0, initial cell value 5·10^6^ CFU/mL). Changes in: a) γ-aminobutyric acid (GABA) concentration (mM), b) cell growth (log CFU/mL), c) pH, mass fractions (mg/g) of acids: d) lactic, e) acetic, f) pyruvic, g) propionic and h) formic were monitored over 18 h. Samples were collected at 0, 3, 6, 9, 12, 15 and 18 h. Data are presented as mean value±standard deviation (*N*=3). Different letters at each time point indicate significant differences (p≤0.05, Duncan's multiple range test)

The primary objective of this study was to optimise GABA production by *L. plantarum* W12 in soymilk. GABA production was monitored throughout the 18-hour fermentation (Fig. 3a). The results show that GABA production is time-dependent. Initially, GABA concentrations were negligible, but they increased steadily, reaching a maximum of (34.5±1.0) mM at 15 h. A slight decrease in GABA concentration was observed at 18 h ((33.5±0.7) mM), although this difference was not statistically significant (p>0.05). The increase in GABA production correlates with the growth of *L. plantarum* W12 and the decrease in pH. This suggests that active bacterial metabolism and the acidic environment created by organic acid production are favourable for GABA biosynthesis (*46*).

*L. plantarum* W12 reached a GABA concentration of 9.1 mM under optimal conditions and demonstrated strong acid and bile resistance, high hydrophobicity, and antimicrobial activity, supporting its suitability for functional food applications. The observed pH decrease (from ~6.0 to 4.6 over 18 h) reflects active metabolism and organic acid production, notably lactic acid, which contributes to preservation and sensory properties (*47*). At 15–18 h, W12 entered the stationary phase with maximum GABA accumulation. GAD activity, optimal at low pH, may be suppressed as GABA production increases at medium pH. Mutant GAD enzymes with broader pH activity have been proposed to overcome this limitation (*48*). Recently, Kubota *et al.* (*7*) showed that GABA attenuates ischaemia-reperfusion-induced alterations in intestinal immunity through increased IgA secretion, alpha-defensin-5 expression, and small intestinal superoxide dismutase activity. These findings indicate that *L. plantarum* W12 in our study is a potential probiotic for producing functional foods to enhance intestinal health.

Cell growth is a critical factor in fermentation as it relates to the production of metabolites, including GABA. The growth of *L. plantarum* W12 in soymilk was monitored over 18 h of fermentation (Fig. 3b). The data show a typical growth curve for *L. plantarum* W12 under these conditions. The initial cell density of 6.7 log CFU/mL increased rapidly during the first 12 h, reaching 7.8 log CFU/mL. After 12 h, the growth rate slowed, and the cell density reached 7.9 log CFU/mL by the end of the 18-hour fermentation. This indicates that *L. plantarum* W12 efficiently utilises the soymilk medium supplemented with MSG and sucrose for growth. The observed growth pattern suggests that the bacteria entered the stationary phase after 12 h, due to nutrient depletion or the accumulation of inhibitory metabolites (*49*). It is important to note that the production of GABA is often associated with bacterial growth and metabolic activity (*50*). Therefore, understanding the growth dynamics of *L. plantarum* W12 is crucial for optimising GABA production. The correlation between cell growth and GABA concentration will be further investigated in subsequent analyses. This will help determine the optimal fermentation time for maximising GABA yield while considering the cost and efficiency of the process.

Monitoring pH changes during fermentation is crucial as it reflects the metabolic activity of the bacteria and can influence enzyme activity, including glutamate decarboxylase, which is responsible for GABA production (*51*). The pH of soymilk decreased significantly throughout the fermentation period (Fig. 3c).

Regarding organic acid profiles, mass fractions of lactic, acetic, pyruvic, propionic and formic acid were monitored over the 18-hour fermentation (Fig. 3d–Fig. 3g). Lactic acid (Fig. 3d) was the dominant organic acid produced, consistent with the metabolic activity of *L. plantarum* W12, a lactic acid bacterium. Its concentration increased steadily throughout fermentation, reaching a maximum of (182.4±7.9) mg/g at 18 h. This progressive increase in lactic acid is directly related to the observed decrease in pH. Acetic acid (Fig. 3e) was produced at much lower mass fractions than lactic acid, reaching approx. 1.10 mg/g by the end of fermentation. Pyruvic acid mass fraction also increased gradually during fermentation, reaching 0.12 mg/g at 18 h. Pyruvic acid (Fig. 3f) is an important intermediate in various metabolic pathways, including lactic acid fermentation (*52*). Propionic acid (Fig. 3g) mass fractions remained stable throughout fermentation, around 0.25 mg/g. Formic acid (Fig. 3h) production showed a different pattern, with an initial increase followed by a decrease. The highest mass fraction (0.02 mg/g) was observed at 9 h.

Our results showing lactic acid as the predominant byproduct ((182.4±7.9) mg/g) with minor amounts of acetic acid (1.10 mg/g) align with the typical homofermentative metabolism of *L. plantarum*. This metabolic pattern is crucial for both product quality and GABA production efficiency. The high lactic acid to acetic acid ratio (165:1) confirms the homofermentative nature of *L. plantarum* W12, in sharp contrast to heterofermentative LAB such as *L. brevis*, which produce higher ratio of acetic acid alongside lactic acid and generate additional metabolic byproducts, including ethanol and CO_2_ (*53*).

The dominance of lactic acid in our fermented soymilk offers several advantages: (*i*) desirable sensory properties, including mild acidity (pH=4.6) and a clean flavour profile without the sharp, vinegary notes associated with high acetic acid content, (*ii*) effective preservation through controlled pH reduction, inhibiting the growth of spoilage organisms and foodborne pathogens, (*iii*) favourable metabolic environment for GAD enzyme activity, as the enzyme shows optimal activity at pH=4.5–5.5, which is maintained during the mid-to-late fermentation stages, and (*iv*) minimal production of off-flavour compounds, as evidenced by the low mass fractions of other organic acids (pyruvic 0.12, propionic 0.25 and formic acid 0.02 mg/g). These low mass fractions of minor organic acids indicate efficient metabolic flux towards lactic acid production, minimising potentially undesirable flavour compounds and ensuring a clean, acceptable taste profile for the final product (*54*).

The temporal dynamics of organic acid production during fermentation (Fig. 3d–Fig. 3h) showed coordinated metabolic processes: lactic acid accumulation parallelled GABA production and the decrease in pH, suggesting coupling between energy metabolism and glutamate decarboxylation. This relationship is consistent with the GAD-mediated acid resistance mechanism, in which GABA synthesis consumes intracellular protons, helping maintain cellular pH homeostasis during lactic acid fermentation (*55*). The stable low mass fraction of propionic acid and the transient peak of formic acid at 9 h (followed by a decline) indicate active secondary metabolism without accumulation of undesirable metabolites.

## CONCLUSIONS

Among the five *Lactiplantibacillus plantarum* strains examined, *L. plantarum* W12 showed the most promising performance, exhibiting superior probiotic traits and achieving the highest gamma-aminobutyric acid (GABA) production ((34.5±1.0) mM) under optimised fermentation conditions (1.564 mg/mL monosodium glutamate (MSG), 10.93 % sucrose, 15 h at 43 °C). The GABA-enriched fermented soymilk produced with *L. plantarum* W12 constitutes a functional food product with dual benefits: high amounts of bioactive GABA for neurological and cardiovascular health support, and viable probiotic bacteria (>7.9 log CFU/mL) for gastrointestinal health promotion. The optimised fermentation process yields a product with favourable sensory characteristics (pH=4.6, mild lactic acid flavour) and extended shelf life due to organic acid preservation. The homofermentative metabolic profile of W12, characterised by high lactic acid production and minimal off-flavour compounds, ensures both product quality and optimal conditions for GABA biosynthesis by maintaining favourable pH ranges for glutamate decarboxylase activity. These results strongly suggest that *L. plantarum* W12 is an excellent candidate for commercial-scale production of GABA-enriched fermented soymilk. This multifunctional food contains GABA at concentrations exceeding those in most reported plant-based fermented products alongside probiotic mechanisms, positioning this product competitively in the functional food market.

However, to fully assess its safety and health benefits, further *in vivo* studies, including animal models and clinical trials specifically, should evaluate: (*i*) GABA bioavailability through pharmacokinetic studies in animal models to confirm absorption and blood-brain barrier penetration, (*ii*) probiotic persistence and colonisation in human gastrointestinal tract using molecular tracking methods such as strain-specific PCR or whole-genome sequencing, (*iii*) sensory acceptability and shelf-life stability under commercial storage conditions (4 °C, 21 days) including assessment of GABA stability and viable cell counts, and (*iv*) scale-up feasibility and cost-effectiveness analysis for commercial production, including evaluation of substrate costs, fermentation efficiency at industrial scale, and regulatory compliance pathways for health claim substantiation.
